# Cecum to pelvis technique: a simple and autologous solution to prevent postoperative complications in pelvic surgery

**DOI:** 10.1007/s00384-024-04649-0

**Published:** 2024-05-26

**Authors:** Hector Guadalajara, Stacye Michelle Toups, Miguel León-Arellano, Anthony Vizarreta, Damián García-Olmo

**Affiliations:** 1 Department of General and Digestive Surgery, Hospital Universitario Fundación Jiménez Díaz, Reyes Catolicos Avenue, 2. 28040, Madrid, Spain; 2 Department of Radiology, Hospital Infanta Elena, Madrid, Spain

**Keywords:** Empty Pelvis Syndrome, Pelvic exenteration, Abdominal perineal resection, Adhesive small bowel obstruction, Cecum, Pelvic occlusion, Adhesions

## Abstract

**Background:**

Empty Pelvis Syndrome, subsequent to the removal of pelvic organs, results in the descent of the small bowel into an inflamed pelvic cavity, leading to the formation of adhesions and subsequent small bowel obstruction. However, no effective measures have been previously described.

**Objective:**

Describe a simple and autologous solution to prevent “Empty Pelvis Syndrome,” small bowel obstruction, and adhesions by utilizing the cecum to occlude the pelvis.

**Design:**

Mobilization of the right colon to lower the cecum into the pelvic cavity to occlude the superior pelvic ring to some degree and changing the direction of the terminal ileum.

**Settings:**

Hospital Universitario Fundación Jiménez Díaz, Department of General Surgery, Colorectal Service.

**Patients:**

Eight anonymized patients were included in this study, each with varying colorectal pathologies. Patients were above 18 years old.

**Main outcome measures:**

Percent of blockage of the superior pelvic ring produced by the descended cecum recorded in percentage; the amount of small intestine descended past the superior pelvic ring recorded in cm.

**Results:**

The mobilization of the cecum achieved partial occlusion of the superior pelvic ring. The descent of the small bowel beyond this landmark ranged from 0 to 4.9 cm.

**Limitations:**

Given the small number of patients included in this study, these results cannot be generalized to the whole of the population. A bladder emptying protocol prior to CT scans was not implemented, resulting in variations in measurements among patients.

**Conclusion:**

The cecum-to-pelvis technique is a simple method that can serve as an autologous solution to EPS (enteropelvic fistula) and help reduce postoperative complications such as SBO (small bowel obstruction) and adhesions. It is not essential to completely occlude the superior pelvic ring to achieve successful outcomes.

**Supplementary Information:**

The online version contains supplementary material available at 10.1007/s00384-024-04649-0.

## Introduction

The surgical removal of pelvic organs and most importantly the rectum leaves a space in the pelvis which then needs to be filled to avoid the descent of the remaining intestines. If the space that was once occupied by the rectum is not filled, patients run the risk of developing “Empty Pelvis Syndrome” (EPS) which is the empty pelvic space that can lead to fluid accumulations and small bowel adherence to the pelvic floor, thus leading to fistulas or sinus formation, pelvic abscesses, a prolonged ileus, mechanical bowel obstruction, or chronic infection [1].

Many techniques have been developed to prevent EPS in varying situations such as gynecological (hysterectomies), urological (cystectomies), and, in the case of this research, digestive techniques (proctosigmoidectomy, abdominoperineal resection (APR), and pelvic exenteration (PE)). Those techniques include mesh reconstruction, breast prosthesis, myocutaneous flaps, omentoplasty, obstetric balloons, and silicone expanders in which each of these was found to still have considerable postoperative complications (POC) [2–4].

With the many options currently to reduce EPS, SBOs and adhesions, there is still no consensus on an optimal technique. However, the cecum to pelvis technique is a simple technique that can reduce morbidity as an autologous solution. It is an operation that consists of completely mobilizing the right colon to be lowered and occupy the pelvis with the cecum. There are very few articles written on this technique, and very few patients utilized it. It may be particularly beneficial following colo-anal anastomosis takedown, definitive Hartmann procedure, abdominoperineal resection, or pelvic exenteration, as well as in cases where residual pelvic tumor is present.

## Method

**Fig. 1:**
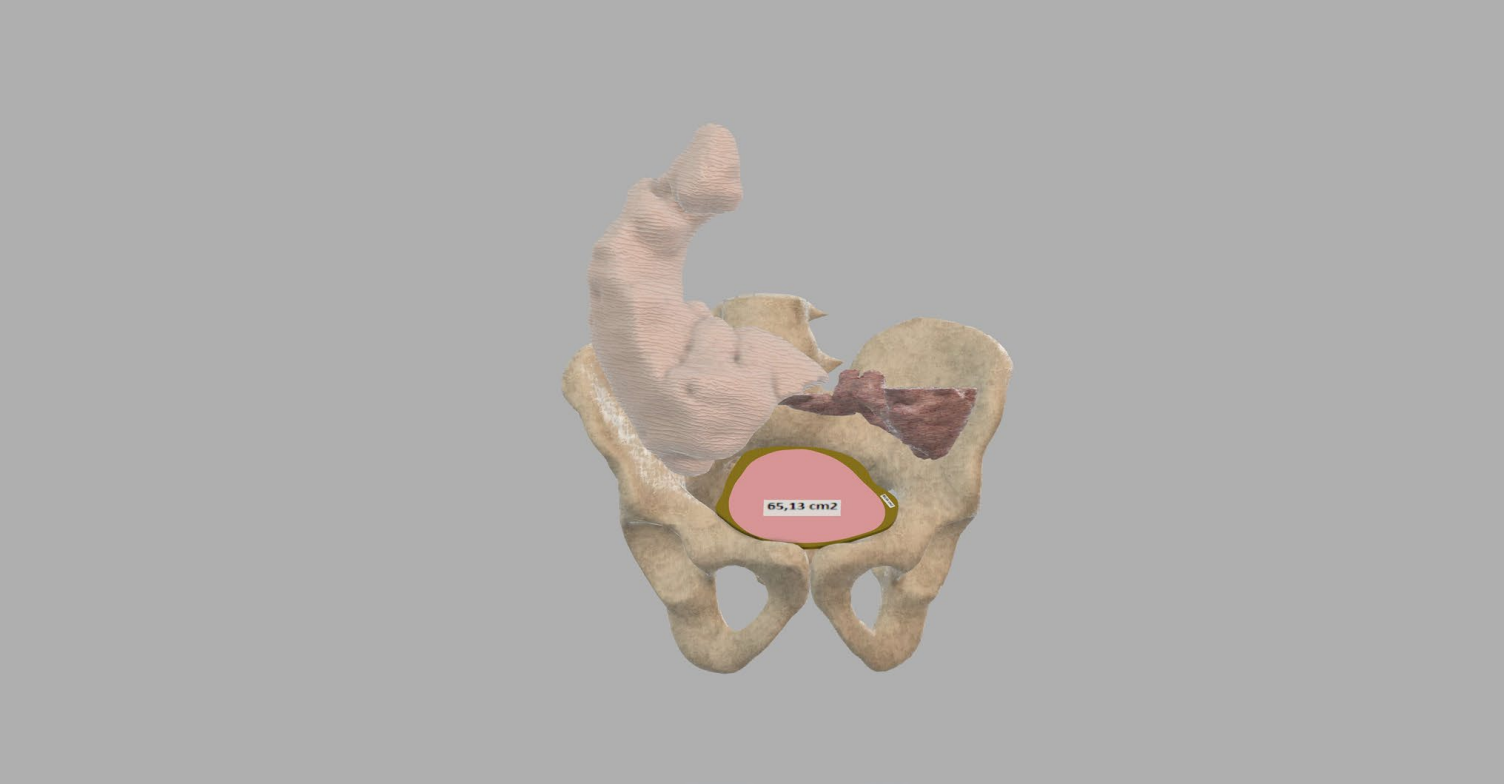
Illustration of the superior pelvic ring

Eight anonymized patients were included that have been treated with the cecum to pelvis technique. They have been reviewed, and our experience in these cases has been described to provide examples of successful cases. The pelvis was defined by the superior pelvic ring (SPR) as a point of reference for the opening to the pelvic cavity. All measurements were based on the SPR which included the percentage of the pelvis occluded by the cecum, bladder, small bowel, and the distance the small bowel has descended into the pelvis (Fig. 1).

### Procedure description

The procedure is straightforward. It begins with a right parietocolic mobilization, moving upwards from the cecum to the hepatic flexure. The right gastroepiploic vein should not be divided; this marks the limit of our dissection. Next, the posterior aspect of the right colon is mobilized, and the right colon is descended to the pelvis to occlude the superior pelvic ring, causing the terminal ileum to take on a vertical trajectory.

Achieving complete occlusion while preserving the right gastroepiploic vein is not always possible, but it is not mandatory. We consider the procedure successful when the terminal ileum assumes a vertical position.

The bladder and/or the uterus may be helpful in achieving a complete obliteration of the superior pelvic ring, but caution must be taken if the bladder is mobilized to avoid denervation.

## Results

### Case 1: adenocarcinoma of the rectum

A robotic Hartmann procedure was performed due to advanced low rectal cancer. Following this procedure, the right colon was mobilized. Postoperatively, the patient presented with a presacral collection 6 days post-op, which was treated with ambulatory care. No other postoperative complications (POCs) such as SBO have been observed.

### Case 2: epidermoid carcinoma of the anus

The patient underwent a robotic abdominoperineal resection during which a vertical rectus abdominis myocutaneous (VRAM) flap was utilized to cover the perineal wound. Subsequently, the right colon was mobilized and descended into the pelvic cavity, positioned above the VRAM flap. Two months postsurgery, the patient presented with a small presacral collection, which was managed through ambulatory care. No other postoperative complications (POCs) have been reported.

### Case 3: enteroperineal fístula

A patient with an enteroperineal fistula following abdominoperineal resection underwent distal jejunal resection followed by a T-T anastomosis. Subsequently, the right colon was mobilized. A dehiscence of the anastomosis after transposition of the cecum was reported as well as a perineal abscess treated with intravenous antibiotics. However, no complications were reported concerning the cecum occluding the pelvis.

### Case 4: pelvic T4 tumor with residual disease with an open rectal stump

This patient presented with a perforated rectum due to previously untreated T4 rectal cancer. In an emergency operation, the Hartmann technique was performed, leaving an open rectal stump due to the inability to perform a complete resection of the tumor. Both the bladder and cecum were lowered to occlude the pelvis. No postoperative complications (POCs) were reported.

### Case 5: persistent pelvic sinus after low anterior resection

The first intervention for this patient involved a low anterior resection followed by T-T anastomosis. A second intervention was then conducted due to a persistent pelvic sinus. A Hartmann technique was performed, with the small bowel also freed from attachment to the pelvis. Subsequently, the cecum was mobilized. In a third intervention, intra-abdominal adhesions outside of the pelvis were reported and removed laparoscopically. However, no postoperative complications (POCs) or intra-pelvic adhesions were reported following the cecum-to-pelvis technique, with improvement noted in presacral and perirectal stump collections.

### Case 6: rectal cancer with local relapse

The patient underwent an abdominoperineal resection, during which the pelvis was not occluded, resulting in the descent of the small intestine into the pelvic cavity and subsequent adhesions of the terminal ileum to the pelvic floor. In a second intervention, the terminal ileum was freed from the adhesions, followed by the liberation of the cecum associated with bladder mobilization into the pelvic space. Postoperatively, a retrovesical hematoma and urinary incontinence due to vesical denervation were reported.

### Case 7: pelvic cloaca

A patient with adenocarcinoma of the middle rectum underwent robotic lower anterior resection. Postoperatively, the patient experienced an anastomotic leakage, resulting in a pelvic cloaca. A second operation was performed due to the development of low anterior resection syndrome. This surgery involved blunt dissection of the pelvic inflammatory plastron and complete disconnection of the previous colorectal anastomosis. Subsequently, the cecum was mobilized and lowered into the lesser pelvis to occlude it and create a blockage for the pelvic cloaca. No postoperative complications (POC) were reported.

### Case 8: transanal rectal tumor resection

Surgery was performed to remove the rectum of a patient diagnosed with rectal adenocarcinoma. A colostomy was performed, along with the descent of the right colon and hepatic flexure to cover the pelvic defect with the cecum. No postoperative complications (POC) were reported.

The summarized results are reflected in Table 1.

**Table 1:** Occlusion of the superior pelvic ring and descend beyond it

	Bladder occlusion (%)	Cecum occlusion (%)	Small bowel occlusion (%)	Descended small intestine (cm)	Complications	Follow up in months
Case 1	44	0	9	4.90	Presacral collection	14
Case 2	19	27	6	1.19	Presacral collection	15
Case 3	56	9	1	1.48	Dehiscence of anastomosis and perineal abscess	33
Case 4	13	17	1	0.3	None	27
Case 5	80	0	0	0	Intra-abdominal adhesions	30
Case 6	0	54	0	0	Vesical denervation	24
Case 7	17	40	2	2.37	None	12
Case 8	47	0	3	2.38	None	11

## Discussion

In several patients, we have successfully implemented this technique, finding that it is not always necessary to fully descend the cecum into the pelvis to achieve the desired result of pelvis occlusion. Even with partial descent of the cecum into the pelvis, positive results can be achieved. The rationale behind this is that changing the direction of the terminal ileum upwards, combined with partial occlusion, will effectively prevent contact with the inflamed pelvis.

TIP 1: Changing the axis of the small intestine, combined with partial pelvic occlusion, reduces the area of small bowel in contact with the inflamed pelvis.

We have found that using the cecum to occlude the pelvis does not completely prevent all pelvic collections that appear below the cecum in cases where it does not fully block the pelvis. However, it does prevent contact of the small intestine with the inflamed area, thereby minimizing the occurrence of EPS. The reason for this may be that some empty space remains, usually forming a residual cavity over the rectal stump, which may lead to chronic pelvic fluid accumulation. In such cases, it may be useful to perform a wide sphincterotomy if the pelvic floor was spared, as it can act as a non-exit valve.

TIP 2: We have also found that the descended cecum can be used in combination with a flap to cover the pelvic floor before occluding the cavity, resulting in beneficial outcomes.

It could also be necessary to include proximal anastomosis when it is performed during the same procedure as a criterion to not use this technique, as the descent of the cecum with the ileum can increase the pressure within the small intestine and create a leak close to the previous anastomosis, as seen with one of our patients. TIP 3: Caution must be taken if the patient has a proximal small bowel anastomosis.

TIP 4: We have also found that using both the bladder and the cecum technique can be redundant in pelvis occlusion and may lead to bladder complications such as bladder incontinence due to denervation that may occur during mobilization. Nonetheless, under certain circumstances, the bladder and/or the uterus may also be helpful in pelvis occlusion. 

We can also argue that while descending, the cecum can prevent intra-pelvic adhesions of the small intestine to the pelvic wall; it does not prevent other types of complications such as intra-abdominal adhesions. TIP 5: This technique does not prevent other types of adhesions.

## Conclusion

The cecum-to-pelvis technique has been demonstrated as a simple method that can serve as an autologous solution to enteropelvic fistula syndrome and reduce postoperative complications, thereby decreasing morbidity in patients undergoing proctosigmoidectomies, abdominoperineal resections, and pelvic exenteration. While this technique does not prevent pelvic abscess formation, it does prevent the small intestine from coming into contact with the inflamed area. Complete occlusion of the superior pelvic ring is not necessary to achieve successful results.

## Supplementary Information

Below is the link to the electronic supplementary material.

Supplementary file1 (MP4 8891 KB)

## Data Availability

No datasets were generated or analyzed during the current study.

## References

[1] Manzour N, Boria F, Chiva L (2021) Empty pelvis syndrome: is there an easy way? Int J Gynaecol Cancer 32(1):114–124 10.1136/ijgc-2021-003196 34725204

[2] Johnson YL, West MA, Gould LE, Drami I, Behrenbruch C, Burns EM et al (2021) Empty pelvis syndrome: a systematic review of reconstruction techniques and their associated complications. Colorectal Dis 24(1):16–26 34653292 10.1111/codi.15956

[3] Micha JP, Goldstein BH, Rettenmaier MA, Brown JV (2004) Cecal pelvic transposition following total pelvic exenteration. Gynecol Oncol 94(2):589–592 15297211 10.1016/j.ygyno.2004.05.008

[4] Guimarães GC, Baiocchi G, Rossi BM, Ferreira FO, Aguiar S, Nakagawa WT et al (2007) The use of silicone expander and cecal transposition after pelvic exenteration. Eur J Surg Oncol 33(5):586–589 17360143 10.1016/j.ejso.2007.01.026

