# Correction to: Cecum to pelvis technique: a simple and autologous solution to prevent postoperative complications in pelvic surgery

**DOI:** 10.1007/s00384-024-04664-1

**Published:** 2024-06-10

**Authors:** Hector Guadalajara, Stacye Michelle Toups, Miguel León‑Arellano, Anthony Vizarreta, Damián García‑Olmo

**Affiliations:** 1 Department of General and Digestive Surgery, Hospital Universitario Fundación Jiménez Díaz, Reyes Catolicos, Avenue, 2. 28040, Madrid, Spain; 2 Department of Radiology, Hospital Infanta Elena, Madrid, Spain

**Correction to: International Journal of Colorectal Disease**

10.1007/s00384-024-04649-0

The Funding is missing in the original published proof.

